# A Novel Method to Determine the Cause of Left Internal Mammary Artery Instant Non-Patency Based on Transit Time Flow Measurement

**DOI:** 10.3389/fphys.2022.901280

**Published:** 2022-06-30

**Authors:** Boyan Mao, Yue Feng, Mengyao Duan, Yihang Dong, Gaoyang Li, Bao Li, Jincheng Liu, Yuting Guo, Minghui Wei, Zhou Zhao, Youjun Liu

**Affiliations:** ^1^ School of Life Sciences, Beijing University of Chinese Medicine, Beijing, China; ^2^ Department of Biomedical Engineering, Faculty of Environment and Life, Beijing University of Technology, Beijing, China; ^3^ Medical Equipment Department, Peking University First Hospital, Beijing, China; ^4^ Institute of Fluid Science, Tohoku University, Sendai, Japan; ^5^ Cardiac Surgery Department, Peking University People’s Hospital, Beijing, China

**Keywords:** computational fluid dynamics, multiscaled model, coronary artery bypass grafting, graft patency, lumped parameter model

## Abstract

**Objective:** After coronary artery bypass grafting (CABG) surgery, the main causes of poor instant patency of left internal mammary arteries (LIMAs) are competitive flow and anastomotic stenosis, but how to determine the cause of LIMA non-patency without interfering with the native coronary artery is still a difficult problem to be solved urgently.

**Methods:** In this study, a 0D-3D coupled multiscaled CABG model of anastomotic stenosis and competitive flow was constructed. After calculation, the flow waveform of the LIMA was extracted, and the waveform shape, common clinical parameters (average flow, PI, and DF), and graft flow FFT ratio results (F0/H1 and F0/H2) were analyzed.

**Results:** For LIMA, these three common clinical parameters did not differ significantly between the anastomotic stenosis group and competitive flow group. However, the waveform shape and FFT ratio (especially F0/H2) of the competitive flow group were significantly different from those of the anastomotic stenosis group. When the cause was competitive flow, there was systolic backflow, and F0/H2 was too high (>14.89). When the cause was anastomotic stenosis, the waveform maintained a bimodal state and F0/H2 was in a normal state (about 1.17).

**Conclusion:** When poor instant patency of the LIMA is found after CABG, the causes can be determined by graft flow waveform shape and F0/H2.

## 1 Introduction

Coronary artery bypass grafting surgery (CABG) is a common method to cure myocardial ischemia. The graft bypasses the stenosis and maintains blood supply to the distal end of the coronary artery, significantly reducing the risk of myocardial ischemia (Beck, 1935; Vineberg and Jewett, 1947; Favaloro et al., 1971). In CABG, left internal mammary arteries (LIMAs) have become the most commonly used graft material due to their excellent long-term patency. The most common anastomosis way is the LIMA to the left anterior descending (LAD) branch. However, LIMA grafts sometimes have poor instant patency due to their own properties and hemodynamics.

When all grafts are anastomosed, the graft patency is called instant patency. When the instant patency is poor, it will affect the blood supply and even cause the graft occlusion in a very short term after the operation. There are two main causes for poor graft instant patency: one is due to anastomotic stenosis and the other is due to competitive flow (Iii and Blackstone, 2008; Nikolaos et al., 2011). Anastomotic stenosis is mainly caused by errors during the operation, and its patency can be improved after the graft is removed and re-anastomosed. However, when the native coronary artery’s stenosis is not serious, some blood can still flow through it, and this blood flow through the native coronary artery is called competitive flow. For LIMA grafts, competitive flow reduces the blood flow within the graft and, in severe cases, leads to the string phenomenon (Seki et al., 1992; Villareal and Mathur, 2000; Beijk and Harskamp, 2013; Halfwerk et al., 2021). At present, the method to determine the occurrence of competitive flow is to clamp the native coronary artery and measure graft flow again. If there is a significant increase in the graft flow, it indicates that competitive flow is significant. However, clamping the native coronary artery can be harmful to the patient, causing plaque to detach and block the distal coronary artery. Therefore, the clinical application of this method is greatly limited.

When the graft is considered to be of poor instant patency, it needs to be repaired. Usually, the surgeon removes the graft and performs the surgery again, which can improve the blood flow in some of the grafts (caused by anastomotic stenosis) but not in the others (caused by competitive flow). Re-performing this surgery arbitrarily not only does not help improve the graft flow but also increases the surgery risk. Therefore, it is an urgent problem to determine the cause of graft instant non-patency.

In clinical practice, transit time flow measurement (TTFM) technology is usually used to obtain the graft flow waveform and determine the graft’s instant patency (P Malagón et al., 2020; Quin et al., 2020; Stastny et al., 2021). TTFM is based on the theory of ultrasonic velocity measurement, and its probe mainly includes an ultrasonic transmitter, reflection plate, and ultrasonic receiving device. It calculates the flow in the graft by measuring the time between the ultrasonic transmitting device and the ultrasonic receiving device. After CABG and before chest closure, surgeons will use a TTFM probe to measure the flow waveform of each graft, determine the graft patency according to the waveform, and decide whether to repair it. In general, three parameters in the waveform are often used to help surgeons assess graft patency: Average flow, pulsatility index (PI), and diastolic velocity–time integral fraction (DF). Low average flow, high PI, and low DF mean a higher risk of instant graft patency. However, no scholar has studied how to classify the specific causes of the poor graft instant patency. Therefore, in order to study this problem, this study chose the modeling and simulation method of CABG to simulate the graft waveform under different instant patency conditions and find the characteristics of the graft waveform that can determine competitive flow or anastomotic stenosis.

## 2 Methods

### 2.1 The CABG Multiscale Model

#### 2.1.1 Reconstruction of the 3D CABG Model

**FIGURE 1:**
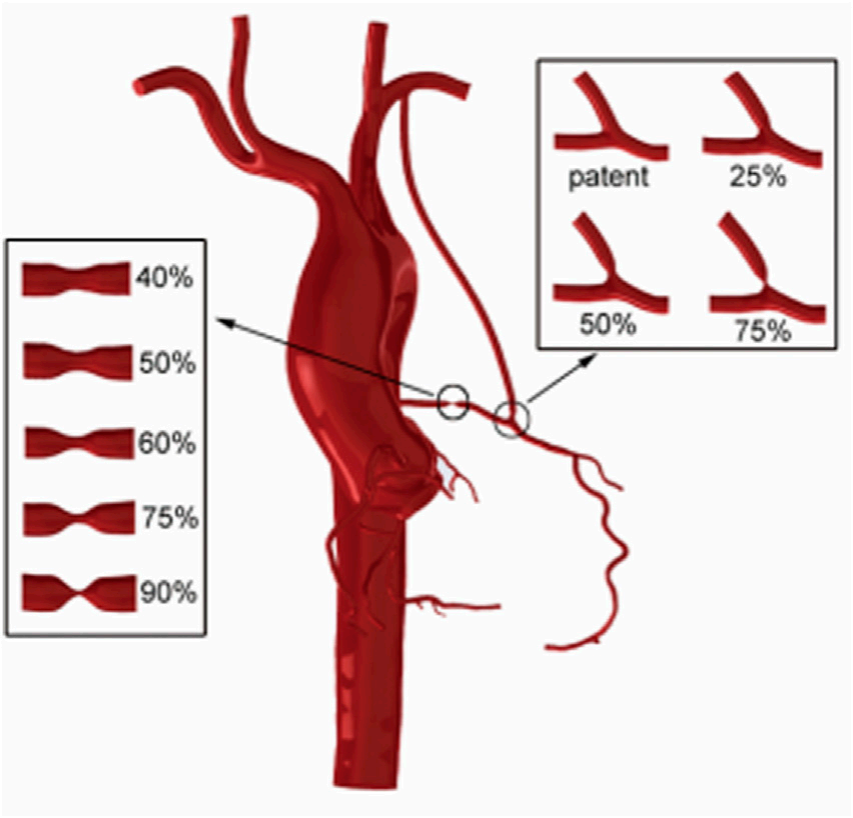
CABG models using the LIMA graft which contain models with different LAD stenosis and different anastomotic stenosis.

This study reconstructed a 3D operation model of the coronary artery system and aortic arch from a patient’s CT image data. The data were obtained from a male 55-year-old patient, and it contained 460 layers in total, each layer had 512*512 pixels, and the space between layers was 1 mm. The patient’s cardiac output was 4.6 L/min, measured by Doppler ultrasound. Also, his diastolic and systolic blood pressures were 103 and 147 mmHg, respectively. A software named “Mimics” was used to conduct reconstruction of CT image data and used threshold segmentation to distinguish the aortic and coronary artery sections. “Freeform” was used to smooth models and complete LIMA-LAD bypass surgery. The LIMA diameter was set as 3 mm. Based on the LIMA-LAD bypass method, the stenosis rates in LAD were set as 90, 75, 60, 50, and 40% to simulate different degrees of competitive flow. In this competitive flow group, the degree of competitive flow increases with the decrease of the LAD stenosis (Li et al., 2017). Also, stenosis in anastomosis was set as 25, 50, and 75% based on a 90% stenosis in LAD (competitive flow is non-significant in this stenosis rate) for the anastomotic stenosis group. Finally, there are eight reconstructed 3D models which are shown in Figure 1. Figure 1 shows the LIMA-LAD models.

“ANSYS-ICEM” was used in meshing these CABG models. All models adopted the tetrahedral meshing method and passed the grid sensitivity analysis. The vessel was assumed to be rigid walled, and the blood flow was a Newtonian fluid, with a dynamic viscosity of 0.0035 Pa s and a density of 1050 kg/m^3^.

#### 2.1.2 Lumped Parameter Model Construction

The lumped parameter model (0D model) used in this work was proposed by Taylor et al. (Taylor et al., 2013), and has been proved to be effective by our previous work (Zhao et al., 2016; Li et al., 2021). The lumped parameter model used a circuit network to simulate the vascular system, and blood flow problem was simplified to a circuit solution problem.

**FIGURE 2:**
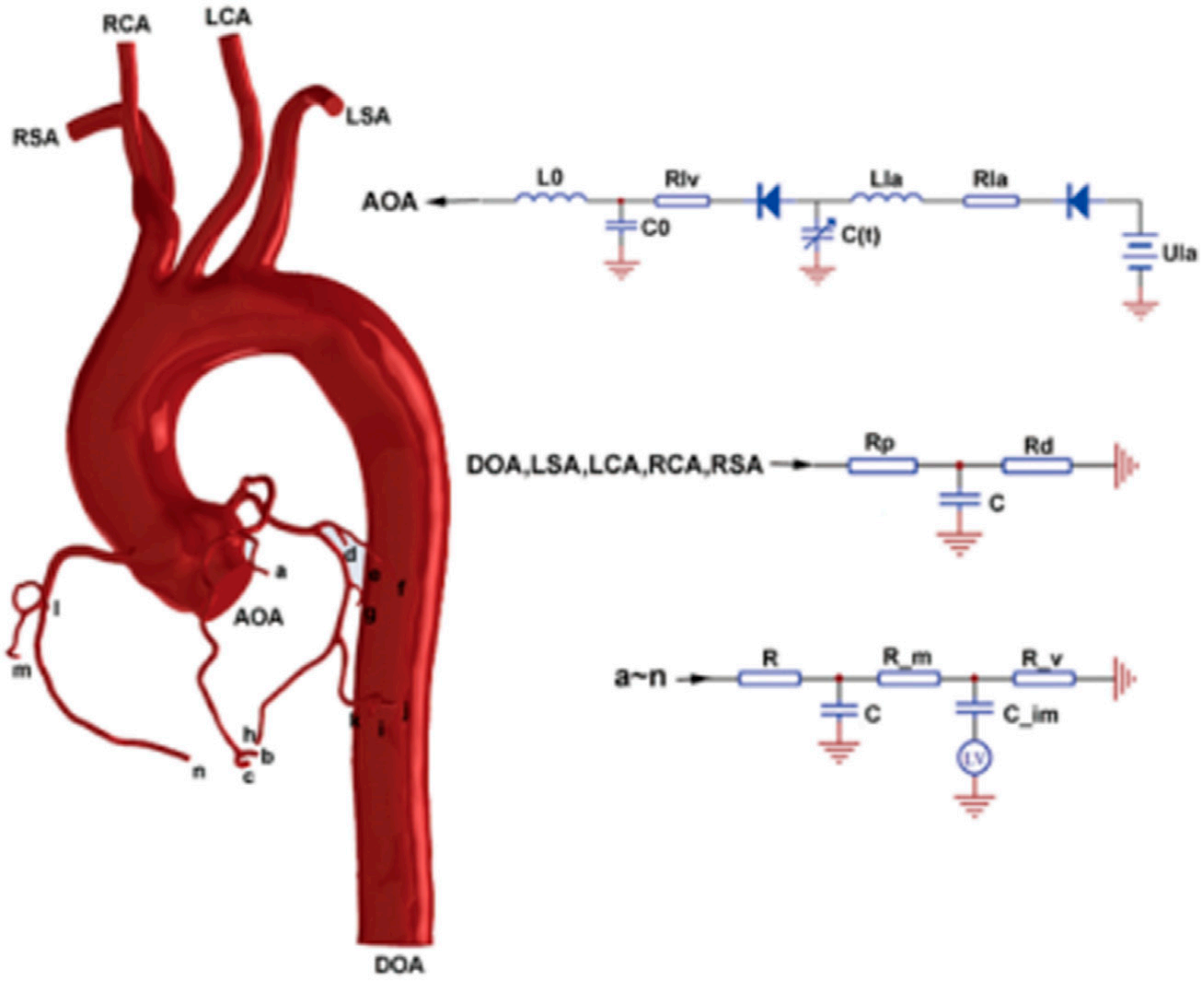
0D/3D coupled multiscaled model.

In this work, the lumped parameter model consisted of the following three modules: the heart module, the aorta module, and the coronary module, as seen on the right side of Figure 2.

For the heart module, a constant voltage power supply Ula was used to represent the pressure of the left atrium, and two diodes were used to simulate the mitral valve and aortic valve in turn. Rla and Lla were used to simulate the blood flow resistance and blood flow inertia flowing through the mitral valve in turn. Time-varying capacitance C (t) was used to represent the relaxation and contraction of the left ventricle. The formula is as follows:

$$\text{C}\left(\text{t}\right)=\frac{1}{\text{E}\left(\text{t}\right)},$$ (1)

where E (t) is a time-varying elasticity (mmHg/ml), which could be approximated as follows:

$$\text{E}\left(\text{t}\right)={\text{E}}_{\text{n}}\left({\text{t}}_{\text{n}}\right)\cdot \left({\text{E}}_{\text{max}}-{\text{E}}_{\text{min}}\right)+{\text{E}}_{\text{min}}.$$ (2)

${\text{E}}_{\text{n}}\left({\text{t}}_{\text{n}}\right)$ is the time-varying elasticity after normalization (Stergiopulos et al., 1996).

$${\text{E}}_{\text{n}}\left({\text{t}}_{\text{n}}\right)=1.55\cdot \left[\frac{1}{1+{\left(\frac{{\text{t}}_{\text{n}}}{1.17}\right)}^{21.9}}\right]\cdot \left[\frac{{\left(\frac{{\text{t}}_{\text{n}}}{0.7}\right)}^{1.9}}{1+{\left(\frac{{\text{t}}_{\text{n}}}{0.7}\right)}^{1.9}}\right],$$ (3)

where ${\text{t}}_{\text{n}}=\frac{\text{t}}{0.2+0.15{\text{t}}_{\text{c}}}$ and ${\text{ t}}_{\text{c}}$ is the time of one cardiac cycle. For this research, the settings are as follows: ${\text{t}}_{\text{c}}$ = 0.8s, ${\text{E}}_{\text{max}}$ = 2.0, and ${\text{E}}_{\text{min}}$ = 0.002458.

For the aortic module, Rp represented arterial flow resistance; Rd represented the sum of the arterial terminal, microcirculation, and venous resistance; and C represented arterial vascular elasticity.

For the coronary module, R represented the coronary artery blood flow resistance, Rm represented the coronary microcirculation resistance, and Rv represented the coronary vein resistance. C represented coronary artery elasticity, and Cim represented coronary microcirculation elasticity. A voltage source was connected to Cim, and its value followed left ventricular pressure.

After determining the structure of the lumped parameter model, the next task is to select the appropriate parameters for each component of the model. In this article, a genetic algorithm was used to optimize the parameters (Li et al., 2018), and the problem of matching component parameters with physiological data was solved by taking the patient’s personalized physiological characteristic data as the target.

First, the data of systolic blood pressure, diastolic blood pressure, heart rate, and cardiac output of normal people were used to fit the waveform of aortic pressure and cardiac output of normal people as two optimization target waveforms. Based on the research of Kim et al. (Kim et al., 2010), manual adjustment was adopted to adjust the parameter values to the degree that the output waveform matched the target waveform, and the parameters at this time were used as the reference values of subsequent personalized parameters. In this process, two important points should be noted: 1) total coronary flow accounted for 4% of cardiac output and left coronary flow and right coronary flow accounted for 60 and 40% of total coronary flow, respectively; 2) the blood flow of coronary artery branches is proportional to 2.7 power of coronary artery diameter.

Second, the clinical measurements of aortic pressure and cardiac output were taken as the optimization objectives, and sensitivity analysis of the parameters of the lumped parameter model was conducted to find the parameters which had a great influence on the optimization objectives. It is found that the sensitive parameters include left atrial pressure and parameters of ${\text{E}}_{\text{max}}$ and ${\text{E}}_{\text{min}}$ , which determine time-varying capacitance and microcirculation resistance.

Finally, the measured systolic blood pressure, diastolic blood pressure, and heart rate were used to adjust the standard aortic pressure waveform, and then the patient’s personalized aortic pressure waveform was obtained. The pressure waveform was compared with the pressure waveform calculated by the lumped parameter model, and the root mean square error between the two waveforms was obtained. The root mean square error between the aortic pressure waveform and the simulated waveform and the mean cardiac output were used as the objective function to optimize the sensitive parameters in the model.

#### 2.1.3 0D/3D Coupled Multiscaled Modeling Method

A 3D model was coupled with a lumped parameter model using a coupling algorithm and an interface condition. ANSYS-CFX commercial software was used for the 0D-3D coupling calculation. In this study, the lumped parameter model was used to provide boundary conditions for the 3D model, instead of directly calling the existing functions in the software as the boundary conditions, so the secondary development of CFX was needed. The secondary development of CFX is a user-defined subroutine based on the FORTRAN language. This study used FORTRAN language to write a subroutine based on CFX specification to calculate the lumped parameter model. The subroutine can assign an initial value to the model, calculate the lumped parameter model, transfer data between the 3D model and the lumped parameter model, coordinate multiprocess calculation, and calculate the hemodynamic parameters not included in CFX. All subroutines can be used in CFX calculation in the form of User CEL Function and User Junction Box Routine.

The lumped parameter model provided a flow boundary condition at the aortic inlet and pressure boundary conditions at the artery outlets of the 3D model. After fluid calculation, a pressure value was returned at the aortic inlet, and flow values were returned at artery outlets so as to facilitate the calculation of the lumped parameter model. ANSYS-CFX was used in 3D model calculation, and FORTRAN subroutines were used in lumped parameter model calculation.

The construction of a CABG multiscaled model was then completed, as shown in Figure 2. The blood flow waveform of the mid-side of the graft calculated by each model was extracted for subsequent analysis.

### 2.2 The Data From Flow Waveform

The data extracted from the flow waveforms for this research included the average flow, diastolic velocity–time integral fraction (DF), pulsatility index (PI), and a frequency index called the fast Fourier transformation (FFT) ratio. The FFT method is a classical signal processing method, which has been used to analyze the graft flow waveform. Takami et al. creatively proposed that FFT results of TTFM can be used as an indicator to determine the graft’s patency. Researchers performed FFT transformation on the TTFM waveform, calculated the ratio of the fundamental wave to the first harmonic wave, took whether the ratio was higher than 1 as the distinguishing value to determine whether the graft was patent, and proposed that the FFT ratio had more accurate distinguishing ability than traditional parameters such as average flow, PI, and backflow rate (Takami and Ina, 2001). Later, Une et al. studied whether the FFT ratio of TTFM waveform would be different among different grafts of target coronary arteries. Also, whether the FFT ratio is more accurate than TTFM waveform parameters alone is analyzed to determine the graft patency. After research, both questions have been answered in the affirmative (Une et al., 2011). All these indicate that the FFT ratio has a very good performance in determining whether the graft is patent. Therefore, in this study, we also tried to use the FFT ratio to determine the cause of graft non-patency and observe whether it can play a significant role.

**FIGURE 3:**
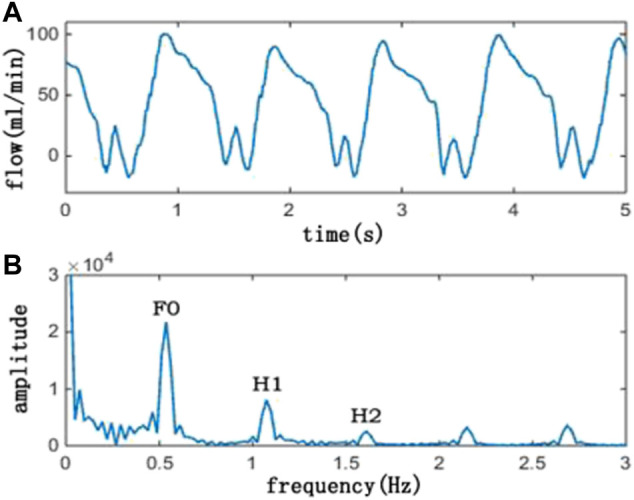
Picture above **(A)** is a TTFM waveform, and the picture below **(B)** is its corresponding spectrum. In the spectrum picture, F0 is the amplitude of the fundamental frequency, H1 is the amplitude of the first harmonic, and H2 is the amplitude of the second harmonic. Also, F0/H1 is the ratio of F0 and H1, and F0/H2 is the ratio of F0 and H2

The average flow was defined as follows: Total flow/time; DF was defined as follows: diastolic flow/total flow; and PI was defined as follows: (maximum flow—minimum flow)/average flow. The FFT method is a classic signal processing method. The TTFM flow measured in the clinic and its spectrum are shown in Figure 3.

In this study, F0/H1 and F0/H2 were selected for research objectives. In order to avoid spectrum leak, cycle extension has been adopted and the flow wave was extended to 10 periods. The sampling frequency was set as 50 Hz, and the sampling point was set as 2048.

## 3 Results

### 3.1 The Comparison of LIMA Graft Flow Waveforms

The flow waveforms have been extracted from the mid-side of the grafts and are shown as the following figures.

**FIGURE 4:**
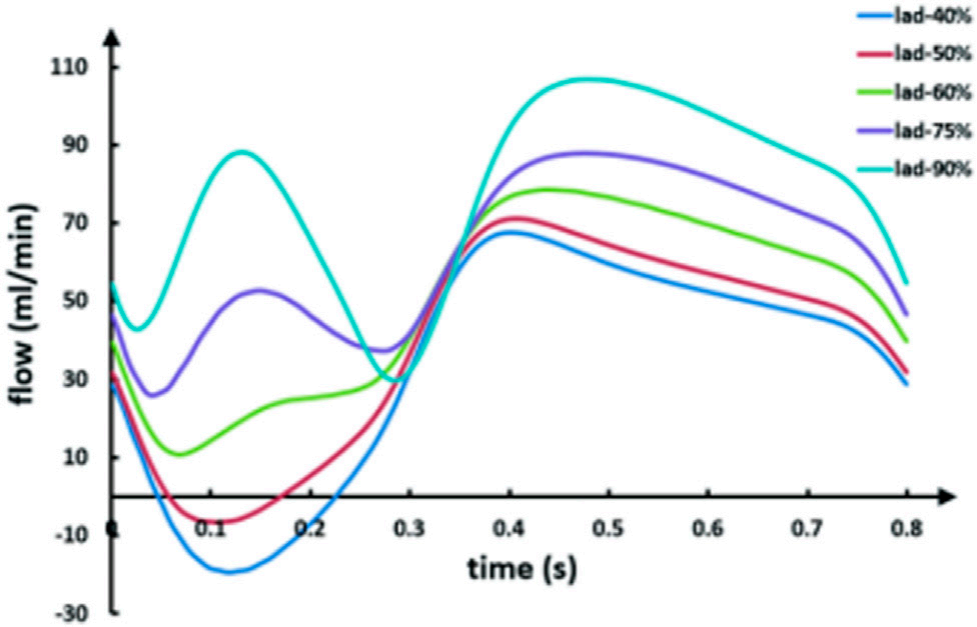
LIMA flow waveforms in models with different degrees of the competitive flow.

Figure 4 shows graft flow waveforms under different LAD stenosis rates. The competitive flow is negatively correlated with the LAD stenosis rate, that is, the lower the LAD stenosis rate, the higher the competitive flow. Therefore, the waveforms shown in the figure are graft flow waveforms under different degrees of competitive flow. It can be seen from the figure that under ideal conditions (i.e., lad-90%, at which the effect of competitive flow can be considered to be very weak), the flow waveform in the graft is a bimodal waveform in systolic and diastolic stages, and the peak value in the systolic stage is slightly lower than that in the diastolic stage. With the increase of competitive flow, the systolic peak flow and diastolic peak flow decrease, but the systolic peak flow decreases more obviously, and the waveform gradually shows a single peak shape. When the LAD stenosis rate is lower than 50%, the peak systolic flow falls below 0 and backflow appears.

**FIGURE 5:**
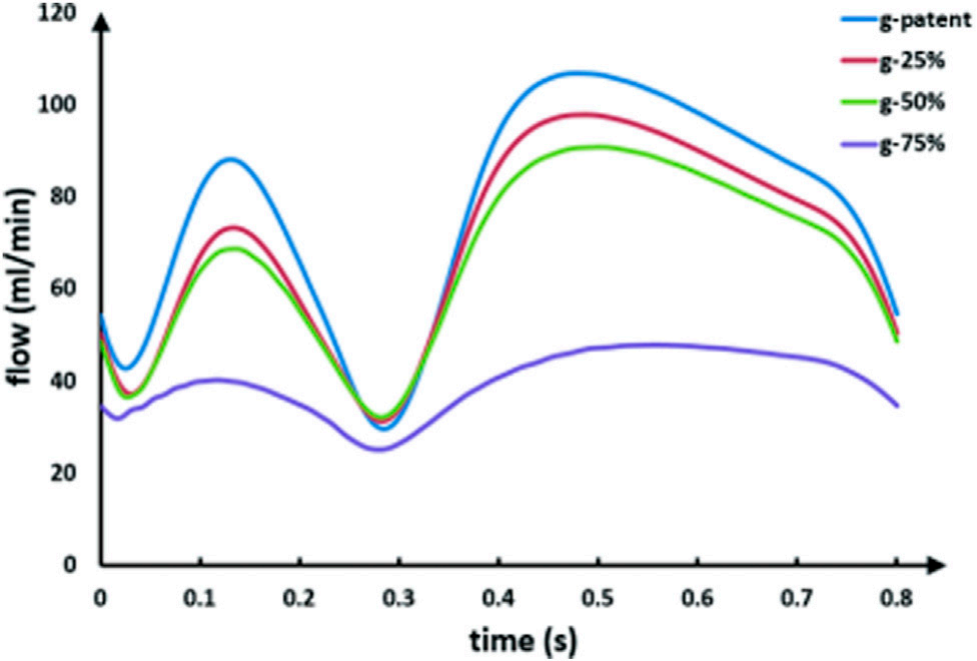
LIMA flow waveforms in models with different anastomotic stenosis based on a 90% LAD stenosis. The g-patent and lad-90% flow waveforms are coming from the same model.

As can be seen from Figure 5, with the increase in the anastomotic stenosis rate, the systolic and diastolic flow in the graft decreases, and the drop scopes are similar. When the anastomotic stenosis rate is 75%, the flow waveform is significantly lower than that of the other three models, but the peak flow ratio of the diastole to systole is basically unchanged. Throughout the process, the waveform maintains a bimodal shape.

In order to quantitatively describe the change of the graft waves, a new index was introduced, which is the ratio of diastolic peak flow to systolic peak flow (D/S). The calculation results are shown in Table 1.

**TABLE 1:** D/S of graft flow waveforms in different models.

Model	D/S
lad-40%	Backflow
lad-50%	Backflow
lad-60%	3.26
lad-75%	1.66
lad-90%/g-patent	1.21
g-25%	1.33
g-50%	1.32
g-75%	1.19

As can be seen from the table, with the LAD stenosis rate changing from 90 to 60%, D/S also increases from 1.21 to 3.26, and systolic backflow even occurs after the stenosis rate is lower than 50%. Also, as the stenosis rate of the anastomotic site changes from the patent to 75%, its D/S remains basically unchanged, ranging from 1.2 to 1.3.

### 3.2 The Comparison of Clinical Factors

Average flow, PI, and DF are commonly used in clinical practice to evaluate the quality of the grafts. They were calculated by extracting graft waveforms. The results are shown in Table 2.

**TABLE 2:** Comparison of clinical factors.

	Average flow (ml/min)	PI	DF (%)
lad-40%	33	2.7	102
lad-50%	39	2	93
lad-60%	50	1.4	83
lad-75%	62	1	75
lad-90%/g-patent	77	1	71
g-25%	70	0.9	72
g-50%	67	0.9	71
g-75%	39	0.6	67

Table 2 shows a comparison of average flow, PI, and DF among different models. As can be seen from the table, for average flow, the maximum flow rate occurs when the LAD stenosis rate is 90% (while the anastomotic site remains patent). As the LAD stenosis rate decreases from 90 to 40%, the average flow rate decreases from 77 ml/min to 33 ml/min. As the anastomosis changes from patent to 75% stenosis, the average flow rate decreases from 77 ml/min to 39 ml/min. For PI, with the decrease of the LAD stenosis rate from 90 to 40%, PI increases from 1 to 2.7. Meanwhile, as the anastomosis changes from patent to 75% stenosis, PI decreases from 1 to 0.6. For DF, DF remains constant in the anastomotic stenosis group, while in the competitive flow group, the LAD stenosis rate reduces from 90 to 40% and DF increases from 70 to 102%. DF reaches 102% due to the presence of systolic backflow, making the total effective flow less than the diastolic flow.

### 3.3 FFT Ratio of Graft Flows

**FIGURE 6:**
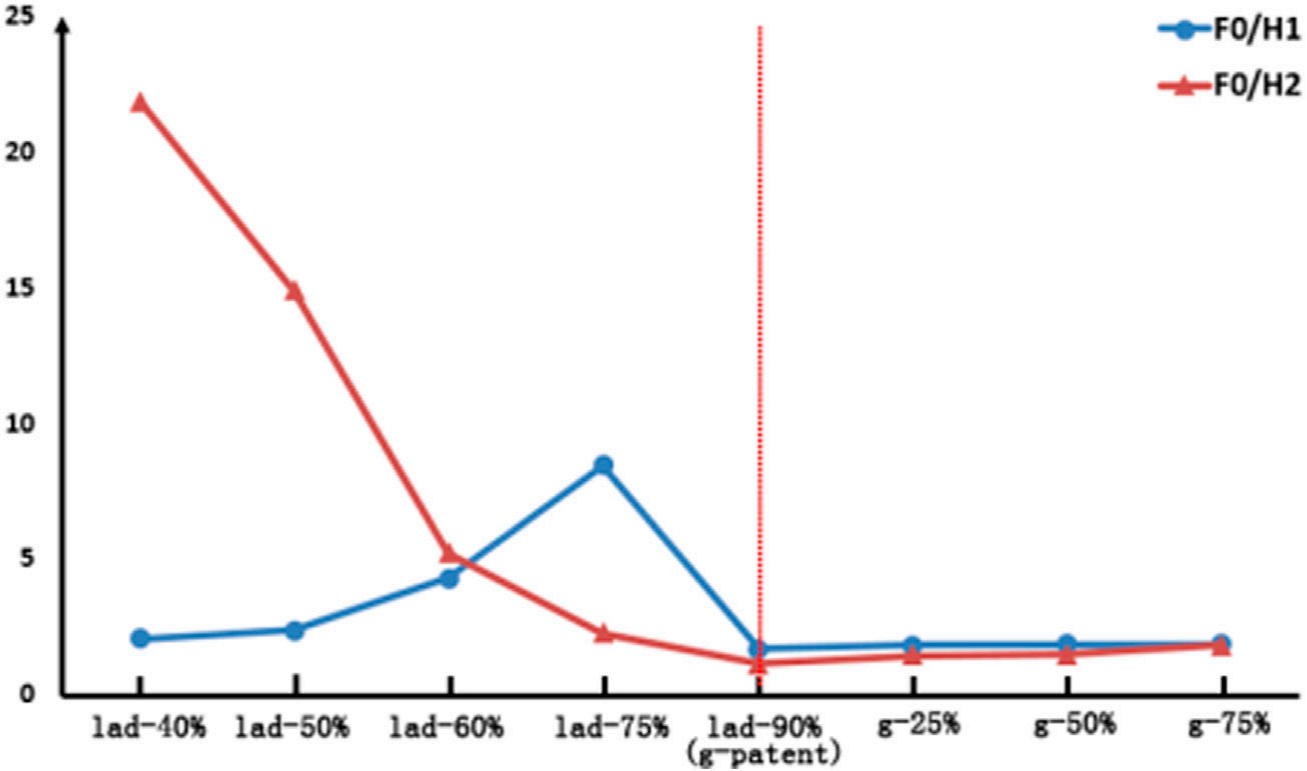
Comparison of the FFT ratio results in LIMA. The left of the red dotted line is the competitive flow group and the right is the anastomosis stenosis group.

By applying FFT transformation to the abovementioned waveforms, F0/H1 and F0/H2 were calculated, and the results are shown in Table 3. To make the results more intuitive, we present the results as line graphs, as shown in Figure 6.

**TABLE 3:** FFT ratio of graft flow waveforms.

	F0/H1	F0/H2
lad-40%	2.07	21.87
lad-50%	2.39	14.89
lad-60%	4.32	5.24
lad-75%	8.44	2.3
lad-90%/g-patent	1.7	1.17
g-25%	1.85	1.45
g-50%	1.89	1.49
g-75%	1.89	1.83

As can be seen from Figure 6, with the LAD stenosis rate reduced from 90 to 40%, F0/H1 first increases from 1.7 to 8.44 but then decreases to 2.07, and the change in this process is nonlinear. However, F0/H2 increases from 1.17 to 21.87. As the anastomotic site changes from patent to 75% stenosis, the values of F0/H1 and F0/H2 both increase, but these changes are not significant.

Based on the abovementioned results, a method to determine the cause of LIMA instant non-patency is obtained in this study. If it is assumed that the stenosis rate of LAD is less than 50%, the competitive flow has a significant effect (Li L et al., 2017). Under this assumption, when the waveform is single-peak and F0/H2 is greater than 14.89, the competitive flow is significant. However, anastomotic stenosis hardly causes the change of D/S and F0/H2, and its D/S is around 1.21 and F0/H2 is around 1.17.

## 4 Discussion

### 4.1 The Variation of Flow Waveforms in Different Models

Based on the graft flow waveforms obtained by our model calculation, waveforms in the competitive flow and anastomotic stenosis groups are significantly different. The change of the competitive flow group is mainly reflected in the shape of the waveform, while the change of the anastomotic stenosis group is mainly due to amplitude. This allows us to use the shape of the waveform as a key factor in determining the cause of graft instant non-patency. To describe the waveform change quantitatively, a new factor D/S is introduced. It can be seen from the results that with the increase of the competitive flow, D/S increases continuously, and even the graft presents backflow. However, as anastomotic stenosis increases, there are minimal changes in D/S. Therefore, D/S could be considered a key determinant factor.

### 4.2 The Analysis of Average Flow, PI and DF

In the LIMA, the ideal model has the maximum average flow. When the competitive flow increases or the anastomotic stenosis increases, the average flow will decrease. This will lead to a challenge in determining the cause using this factor. PI in the competitive flow group is slightly higher than that in the anastomotic stenosis group, and PI increases with the increase of the competitive flow, but it decreases slightly with the increase of the anastomotic stenosis. DF in the competitive flow group increases with the increase of competitive flow, while DF in the anastomotic stenosis group remains constant. However, in general, there is little difference between PI and DF between the two groups, and none of them could be the factor for determining the cause of graft instant non-patency.

### 4.3 The Analysis of FFT Method

After FFT transformation, F0/H1 and F0/H2 have different changing trends. For the anastomotic stenosis group, the change curves of F0/H1 and F0/H2 almost coincide, and the anastomotic stenosis model has no significant change compared with the ideal model. However, for the competitive flow group, not only the model with high competitive flow is very different from the ideal model but also the variation rules of F0/H1 and F0/H2 are different. Compared with the rule that F0/H2 increases monotonically with the increase of competitive flow, F0/H1 is not suitable to be used as a factor in determining the cause of graft instant non-patency due to its non-monotonic characteristics. Therefore, F0/H2 can be considered a factor in determining the cause of LIMA instant non-patency.

### 4.4 Clinical Graft Flow Waveform Results

**FIGURE 7:**
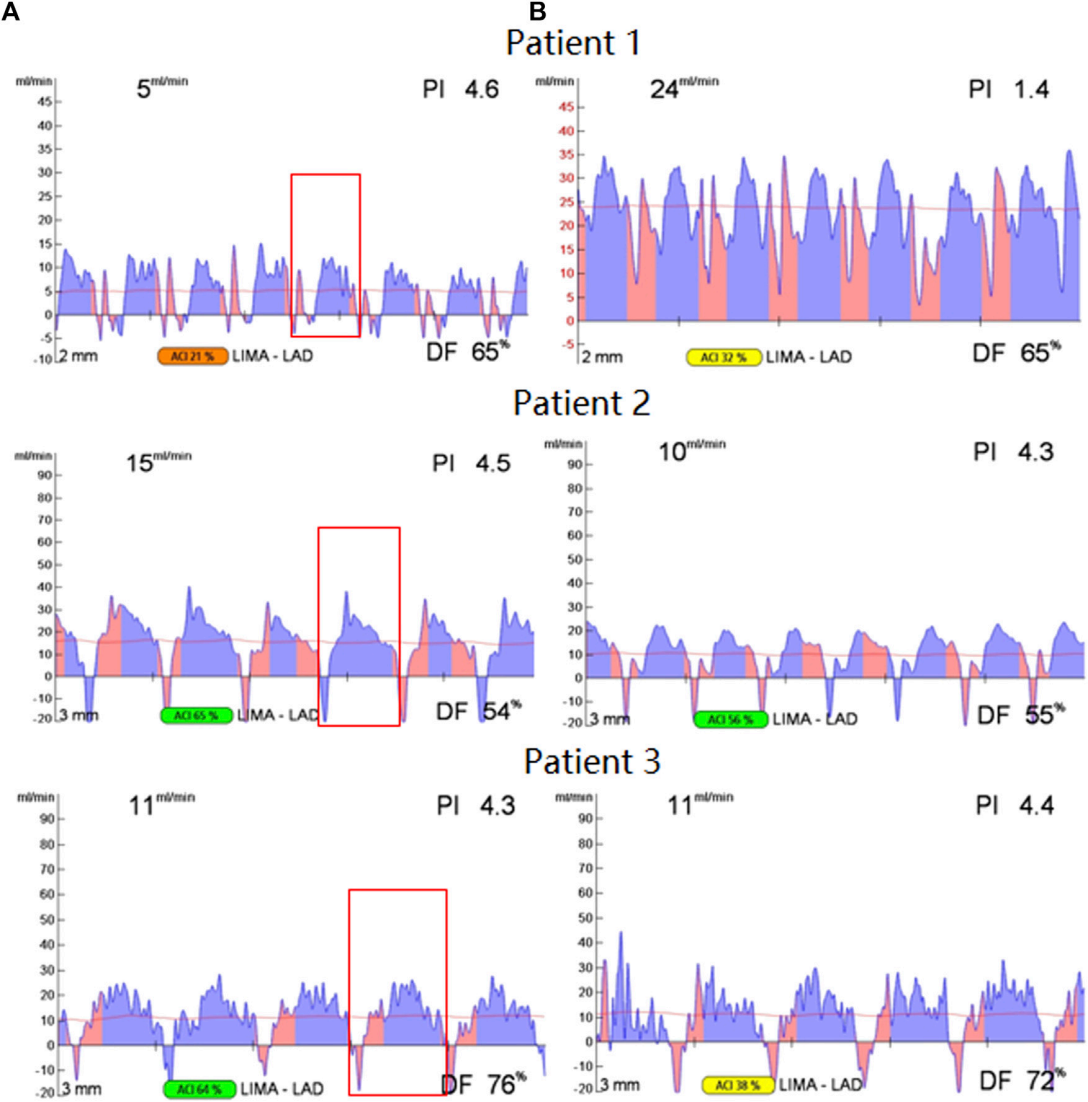
TTFM waveform before and after re-anastomosis, the waveform before re-anastomosis is on the **(A)**, the waveform after re-anastomosis is on the **(B)**, and the selected waveform in the red box of the waveform on the left is for FFT transformation.

In the 132 grafts collected from 60 patients in the previous stage of this study (Mao et al., 2020), a total of 50 LIMA grafts were anastomosed on the LAD branch, and the TTFM waveform of three grafts showed instant non-patency and was re-anastomosed. At present, it is impossible to determine whether the cause of graft instant non-patency is anastomotic stenosis or competitive flow in clinical practice. Therefore, in this study, the grafts with improved flow after re-anastomosis are considered anastomotic stenosis, and the grafts with unimproved flow are considered competitive flow. Based on this principle, one of the three grafts is due to anastomotic stenosis and two are due to competitive flows, as shown in Figure 7.

As can be seen from Figure 7, for patient 1, the graft flow increases from 5 ml/min to 24 ml/min after re-anastomosis, which is a significant increase. Therefore, it is believed that the cause of graft instant non-patency is anastomotic stenosis. However, for patient 2 and patient 3, it could be seen that flows do not increase after re-anastomosis, so it is believed that the graft's instant non-patency is due to competitive flow.

The left TTFM waveform is observed (before re-anastomosis). A period is selected after the flow waveform is stable, and the selected period is in the red box. The period continuation and FFT transformation of this period waveform are carried out. The waveform of patient 1 basically maintains a double peak in systolic and diastolic periods, while waveforms of patient 2 and patient 3 show a single peak in the diastolic period, and there is obvious negative flow in the systolic stage. After the FFT transformation of the selected waveform, the F0/H2 value is obtained. After calculation, the F0/H2 values of patient 1, patient 2, and patient 3 are 1.99, 15.55, and 13.46, respectively. The difference between patient 1 and the remaining two patients validates the method proposed in this study.

The previous method to determine graft competing for flow and anastomotic stenosis was to clamp the native coronary artery and observe whether graft flow was improved. Our method is to directly use the TTFM waveform through the extraction of waveform features to achieve. Comparatively speaking, the method proposed in this article has the greatest advantage of reducing the possible trauma to patients compared with the previous method because clamping the coronary artery can cause plaque to detach and block the distal coronary artery. However, our method avoids this and achieves a “risk-free” judgment of the cause of graft instant non-patency.

### 4.5 Limitation

Although a method to determine the cause of the graft instant non-patency is obtained in this study, there are still some limitations in the experimental process. Among them, the most important point is that the conclusion of this study was reached through modeling and simulation and variable control, and the sample size was also small in the process of clinical verification, so a clear cut-off value for determining competitive flow or anastomotic stenosis could not be obtained. In addition, rigid walls were used in the 3D modeling process, ignoring the influence of vascular elasticity on the waveforms. The fluid-structure coupling model can be considered to solve this problem in future research.

## 5 Conclusion

Based on the abovementioned studies, it is found that for the LIMA graft, when the graft is considered to be non-patent, the waveform shape and FFT ratio results could jointly be used as factors in determining the cause of graft instant non-patency. When backflow occurs during the systolic period and F0/H2 is too high (about 14.89 or higher), it indicates that the cause is competitive flow. When graft flow presents a bimodal state and F0/H2 is at a normal level (about 1.17 or slightly higher), it indicates that the cause is anastomotic stenosis.

## Data Availability

The original contributions presented in the study are included in the article/Supplementary Material; further inquiries can be directed to the corresponding author.
